# Special Issue: Polymers, Polymer Blends, and Polymeric Drug Release Systems with Antiviral or Antibacterial Effect—2nd Edition

**DOI:** 10.3390/ijms262311711

**Published:** 2025-12-03

**Authors:** Piotr Dobrzyński

**Affiliations:** 1 Faculty of Science and Technology, Jan Długosz University in Czestochowa, 13/15 Armii Krajowej Av., 42-200 Czestochowa, Poland; p.dobrzynski@ujd.edu.pl; 2 Centre of Polymer and Carbon Materials, Polish Academy of Sciences, 41-819 Zabrze, Poland

Although the crisis stemming from the global pandemic caused by the coronavirus SARS-CoV-2 seems to have subsided, the threats to global health posed by pathogenic viruses and bacteria remain significant. The experience gained from the fight against COVID-19 indicates that the world remains unprepared for another pandemic caused by pathogens [1]. This situation is aggravated by the issue of increasingly emerging antibiotic-resistant bacteria, commonly known as “superbugs”, which pose a serious threat to human health. These multidrug-resistant strains pose a particular threat to critically ill and hospitalized patients. The World Health Organization (WHO) has identified resistance of bacterial and fungal pathogens to antimicrobial agents as one of the top ten global health threats facing humanity today [2]. In general, approaches to managing infectious diseases include vaccination, the use of antimicrobials, good hygiene practices, safe food handling, and public health measures such as surveillance and forecasting. Due to the decreasing effectiveness of previously employed bioactive substances, the search for new, highly effective antibacterial and antiviral agents has also become crucial. Current research is largely focused on the search for and use of new direct-acting agents that do not exhibit cross-resistance with existing antibiotics [3]. These activities also include the search for methods for the synthesis of new polymers with strong antibacterial and antiviral activity and the increasingly wider use of such polymers and plastics in medicine and healthcare, in addition to the use of new micro- and nanocarriers of antibacterial and antiviral drugs in appropriate therapies as controlled drug release systems, supporting the antimicrobial activity of the released active agent [4].

We are pleased to present the second edition of the Special Issue entitled “Polymers, Polymer Blends, and Polymeric Drug Release Systems with Antiviral or Antibacterial Effect”, which serves as a collection of scientific publications on topics closely related to the aforementioned, ongoing fight against pathogens.

In the edition, the publications focus on methods for synthesizing new polymers that exhibit antibacterial and antiviral activity. They also cover the formation and operation of controlled-release systems for these agents. Musiał et al. (Contribution 1) introduce a novel synthesis route for ring-opening polymerization (ROP) copolymers of ε-caprolactone with the antiretroviral drug lamivudine. The resulting product, which is a form of the lamivudine prodrug, was subsequently used to create microparticles capable of controlled, gradual drug release. Additionally, Śmigiel-Gac (Contribution 2) describes intriguing polymers obtained through stepwise transesterification using endogenous polyamines. These polymers, which contain amide and amine groups in their chain, are linear, non-cytotoxic, hydrophilic block polymers that exhibit strong antibacterial properties against several harmful health strains. The authors of the following paper (Contribution 3) discuss the use of linear or choline-grafted amphiphilic polymers derived from the ionic liquid [2-(methacryloyloxy)ethyl]trimethylammonium chloride (TMAMA_Cl^−^) as nanomatrices for single and dual drug delivery systems of isoniazid, a medication used to treat tuberculosis. The paper authors outline the procedure for creating nanocapsules containing the drug and examine its in vitro release. Additionally, Adamus’ research team (Contribution 4) presents a different controlled-release system utilizing proanthocyanidins extracted from *Pelargonium sidoides* as an active antibacterial agent. This system is designed for local application in dentistry, consisting of nanofibers made from biodegradable polyesters that are placed in tooth pockets. This series of publications is further enhanced by a review work (Contribution 5) in which the authors explore the development of antibacterial chitosan composites using mineral clays and evaluates their potential applications.

Of course, the publications featured in the above Special Issue provide only limited insight into the extensive and complex research surrounding the development and use of polymeric antibacterial materials [4]. While the Issue comprise relatively few papers, they highlight the diverse topics being addressed, which facilitates understanding of the breadth of issues within this research field and the potential opportunities that may arise from their findings. The significance of these investigations is further emphasized by growing interest among scholars, as indicated by a rapid increase in publications on polymers with antibacterial and antiviral properties in recent years. Based on Scopus data, 330 such articles were published in 2010, over a thousand in 2018, and an impressive 2590 in 2024. Unfortunately, this surge in publications has not been matched by a corresponding increase in clinical trials, which remain relatively limited [5,6]. This fact demonstrates that these studies are primarily conducted during the preliminary research phase at present, and their transition into commercial applications is challenging, prolonged, and not entirely straightforward [3,4].
